# Understanding the 2D-material and substrate interaction during epitaxial growth towards successful remote epitaxy: a review

**DOI:** 10.1186/s40580-023-00368-4

**Published:** 2023-04-28

**Authors:** Jongho Ji, Hoe-Min Kwak, Jimyeong Yu, Sangwoo Park, Jeong-Hwan Park, Hyunsoo Kim, Seokgi Kim, Sungkyu Kim, Dong-Seon Lee, Hyun S. Kum

**Affiliations:** 1 Department of Electrical and Electronic Engineering, Yonsei University, Seoul, South Korea; 2 School of Electrical Engineering and Computer Science, Gwnagju Institute of Science and Technology, Gwangju, South Korea; 3 Department of Nanotechnology and Advanced Materials Engineering, Sejong University, Seoul, South Korea; 4 Venture Business Laboratory, Nagoya University, Furo-Cho, Chikusa-ku, Nagoya, 464-8603 Japan

**Keywords:** Remote epitaxy, Heterogeneous integration, Compound semiconductor, Complex-oxide, MOCVD, MBE, PLD, Graphene, TMDC, h-BN

## Abstract

Remote epitaxy, which was discovered and reported in 2017, has seen a surge of interest in recent years. Although the technology seemed to be difficult to reproduce by other labs at first, remote epitaxy has come a long way and many groups are able to consistently reproduce the results with a wide range of material systems including III-V, III-N, wide band-gap semiconductors, complex-oxides, and even elementary semiconductors such as Ge. As with any nascent technology, there are critical parameters which must be carefully studied and understood to allow wide-spread adoption of the new technology. For remote epitaxy, the critical parameters are the (1) quality of two-dimensional (2D) materials, (2) transfer or growth of 2D materials on the substrate, (3) epitaxial growth method and condition. In this review, we will give an in-depth overview of the different types of 2D materials used for remote epitaxy reported thus far, and the importance of the growth and transfer method used for the 2D materials. Then, we will introduce the various growth methods for remote epitaxy and highlight the important points in growth condition for each growth method that enables successful epitaxial growth on 2D-coated single-crystalline substrates. We hope this review will give a focused overview of the 2D-material and substrate interaction at the sample preparation stage for remote epitaxy and during growth, which have not been covered in any other review to date.

**Graphical Abstract:**

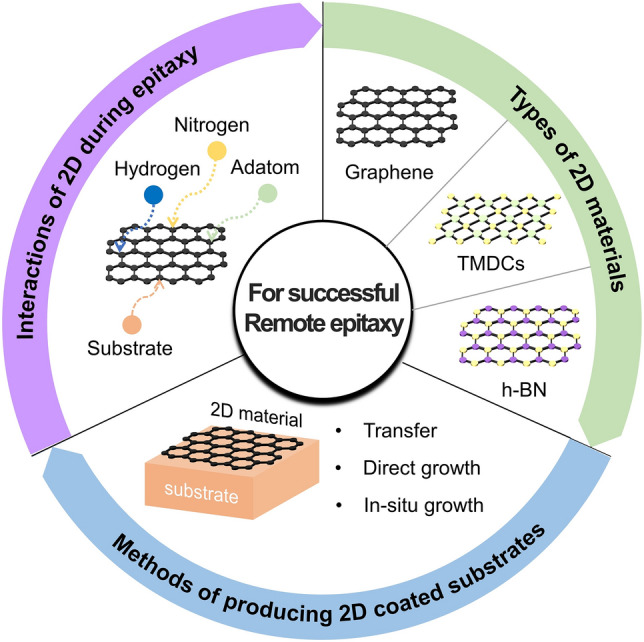

## Introduction

Remote epitaxy is a novel epitaxial technique in which epitaxy is carried out on a single-crystalline substrate coated with an atomically thin two-dimensional material such as graphene, which is composed of a single carbon layer. Since graphene is atomically thin, the surface potential of the underlying single-crystalline substrate can penetrate through the graphene, allowing epitaxial registry to the substrate while simultaneously preventing covalent bond formation between the epitaxial layer that is being grown. This ultimately allows easy peeling of the epitaxial layer off of the 2D material coated substrate, producing ultra-thin and flexible single-crystalline membranes [1, 2].

Remote epitaxy has been demonstrated for a diverse system of single-crystalline material systems such as compound semiconductors (III-V materials, III-N materials, wide band-gap materials) and complex-oxides (perovskites, spinels, and garnets), proving its wide applicability in producing freestanding single-crystalline membranes for desirable functional materials for electronic, photonic, and quantum information system applications. Although remote epitaxy has been experimentally demonstrated by many groups, there is still a lack of complete understanding of the experimental basis in which successful remote epitaxy can be achieved. This is due to the fact that there are several epitaxial growth techniques, such as molecular beam epitaxy (MBE), metal–organic chemical vapor deposition (MOCVD), and pulsed-laser deposition (PLD), which have vastly different growth conditions. These growth conditions (growth pressure, temperature, source type, ambient etc.) greatly affect the coated 2D material, leading to micro and macroscopic damage which ultimately prevents successful remote epitaxy. Moreover, the interaction of source species and surface reconstruction of the substrate surface during growth may also be a factor which prevent successful remote epitaxy.

All of these parameters have not been fully explored experimentally to date, which are critical details that ultimately determine if remote epitaxy is truly a revolutionary discovery that can change the paradigm for the semiconductor device industry, or just another passing hype. Thus, this review serves as a critical guide as to what has been experimentally verified thus far in the context of the interaction of the 2D material coated interface with the substrate during growths in various growth tools and conditions, and what needs to be done to fully understand this interaction.

In this review, first we will briefly introduce the types of 2D materials that is applicable and has been applied to successful remote epitaxy. Next, we will discuss the various methods to fabricate 2D material coated single-crystalline substrates which is one of the key factors for remote epitaxy. Next, we will discuss recent studies that elucidate the interaction between the 2D material and substrate during remote epitaxy under various growth environments, such as MOCVD, MBE, and PLD. Although many experimental works have been published, these works have not been organized nor analyzed systematically thus far. Finally, we will conclude with an overview of successful single-crystalline membrane material systems that have been demonstrated via remote epitaxy.

## Epitaxy on 2D-materials coated substrates

**Fig. 1:**
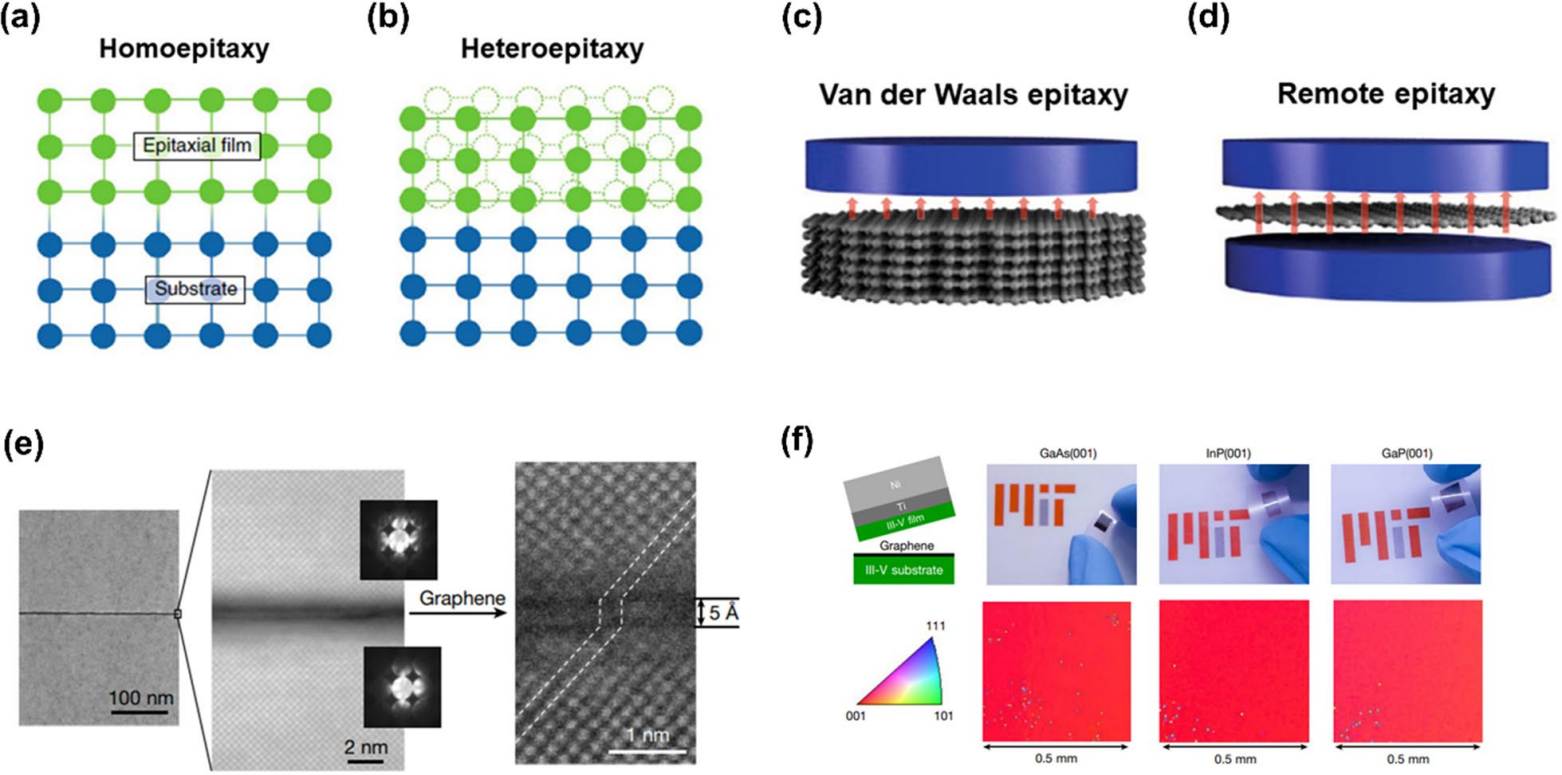
The schematic of epitaxy techniques for crystal growth and demonstraion of remote epitaxy with III-V materials. **a** Homoepitaxy, **b** Heteroepitaxy, **c** van der Waals epitaxy and **d** Remote epitaxy. Figure reproduced from ref, [3, 10], Springer Nature Ltd. **e** High-resolution cross-sectional scanning transmission electron microscopy (STEM) images showing remote epitaxial alignment of the GaAs through the graphene. **f** Single-crystalline III-V membranes exfoliated from graphene/III-V substrates via remote epitaxy. The large-scale electron backscatter diffraction (EBSD) maps below show the single crystallinity of each epilayer. Figure reproduced from ref. [1], Springer Nature Ltd

Epitaxy is a technique for growing single-crystalline films on a crystallographically oriented crystalline substrate following the crystallographic registration of that substrate [3]. There are two categories of epitaxy: homoepitaxy, in which the substrate and epitaxial layer materials are the same (Fig. 1a), and heteroepitaxy, in which they are different (Fig. 1b). A high-quality single-crystalline epitaxial layer can typically be grown using homoepitaxy. However, this can be relatively costly, especially for materials like GaN, due to the scarcity and difficulty of producing large-scale single-crystalline GaN substrates. Therefore, heteroepitaxy is much more common for the growth of device-grade compound semiconductor heterostructures. In contrast, heteroepitaxy usually results in an epitaxial layer with defects such as threading dislocations due to mismatch of the lattice parameter and thermal expansion coefficient between the substrate and the epitaxial layer materials [4]. In order to reduce the dislocation density of the epitaxial layer, several techniques such as metamorphic growth, introducing a thick buffer layer [5], and selective area epitaxy using an additional patterned mask [6], have been reported. However, the quality of the epitaxial layer in large lattice mismatch system is still inferior to that of homoepitaxy. To overcome this challenge, novel approach of epitaxy research through non-direct bonding has emerged [7]. The method of implementing epitaxy with non-direct bonding can be typically divided into van der Waals (vdW) epitaxy and remote epitaxy. Both technologies have something in common to alleviate the difference in lattice mismatch and thermal expansion coefficient by inserting a 2D interlayer, such as graphene, h-BN, between the substrate and the growth material [8, 9]. According to previous reports, vdW epitaxy is classified as having a crystallographic relationship with the interlayer (Fig. 1c), and remote epitaxy is classified as having a crystallographic relationship with the substrate (Fig. 1d) [10]. Additionally, it is possible to create freestanding membranes and reuse expensive wafer by non-destructive exfoliation of the epitaxial layer from the host substrate via 2D material-assisted layer transfer (2DLT).

Remote epitaxy was first reported by Kim et al. [1] in 2017, where an epitaxial layer is grown on a 2D-coated substrate by columbic interaction between an adatom and the underlying substrate, thus epitaxy can remotely occur through 2D material. The potential field of the substrate is not completely screened by a few layers of 2D material, thus the adatom still interacts with the substrate and grow in the same crystal orientation of the substrate. In this technique, it is necessary to form a few layers of 2D material (typically, 1 – 3 layers of graphene) on the substrate through the methods described in Sects. 3 and 4, and then epitaxial growth is performed on the 2D-coated single-crystalline substrate. Firstly, remote homoepitaxy of III-V materials, represented by GaAs, was successfully demonstrated on a monolayer graphene-coated substrate through the growth of a single-crystalline epitaxial layer (Fig. 1e). In addition, it has been reported that the grown epitaxial layer can be easily detached from the substrate non-destructively due to the weak vdW interface of the 2D material (Fig. 1f). Remote epitaxy of III-V materials requires several requirements: sufficiently thin graphene (monolayer) and clean graphene-substrate interface [11, 12]. It was revealed that the polarity of the underlying substrates and the 2D material affects remote interaction through the 2D material, making relatively more strict requirements for remote epitaxy of III-V materials are necessary [13].

## Types of 2D materials applicable for remote epitaxy

In this section, we will briefly discuss the different types of 2D materials and synthesis techniques that can be used for remote epitaxy. There are many other research and review articles which go much deeper into the synthesis and characteristics of these materials [14–16], thus only a brief overview will be given to the materials that are relevant to remote epitaxy, and those who are already familiar with this subject may skip this section completely.

### Graphene

Graphene is formed by a network of carbon atoms bonded with sp^2^ hybridization in a two-dimensional hexagonal lattice, and features unique physical, chemical, and mechanical properties different from those of conventional three-dimensional materials [17, 18]. Therefore, it is a desirable material for applications in high-tech industries such as electronic devices [19–21], optical devices [22] and fuel cells [23, 24]. The synthesis of graphene can be classified into (1) a top-down approach via mechanical and chemical exfoliation from bulk carbon and (2) a bottom-up approach in which graphene is formed on specific metallic, semiconducting, and insulating substrates. Table 1 summarizes various synthesis and transfer methods of two-dimensional graphene for non-conventional epitaxial growth reported to date. We will briefly discuss each method and how it relates to remote epitaxy.

**Table 1:** Various synthesis and transfer methods of graphene

Material	Seed substrates	Synthesis method	Crystallinity	Thickness	Transfer method	References
Graphene	Graphite	Mechanical exfoliation	Single crystal	Variable	Sticky tape	[21, 25–27]
	Graphite oxide	Chemical exfoliation			–	[28–31]
	Cu foil	CVD	polycrystal	Multiple layer	Wet etching	[34–39]
			Single crystal	Single layer	Wet etching	[40]
				Single- to bilayer		[41]
	Ni foil	CVD	Variable	Bilayer	Wet etching	[42–44]
			Single crystal	Single- to few-layer	Wet etching	[45]
		DAS	Variable	Multiple layer	Wet etching	[51, 52]
	Pd	CVD	Single crystal	Monolayer	–	[46]
	Ir					[47]
	Cu-Ni alloy foil	CVD	Single crystal	Monolayer	Wet etching	[49]
				Bilayer Tri-layer		[50]
	Ge	CVD	Single crystal	Variable	Dry transfer	[56]
				Monolayer	–	[57]
						[58]
	c-plane sapphire	CVD	Single crystal	Monolayer	Wet etching	[60]
	SiC	CVD	Single crystal	Monolayer	Ni stressor	[61, 62]
			Single crystal	Monolayer	–	[63, 64]
				Multilayer	–	[65]
		MBE	Single crystal	Monolayer	–	[66, 67]
				Variable	–	[67–69]

#### Top-down approach

Although monolayer graphene mechanically exfoliated from highly oriented pyrolytic graphite (HOPG) with multilayer structure exhibits superior mechanical strength and stable chemical/thermal properties due to high crystallinity [21], advanced approaches for obtaining large-area graphene have been attempted to overcome the low yield caused by the size of the exfoliation tape and the impossibility of accurate thickness control [21, 25–27]. The physically and chemically exfoliated graphene sheets form a large quantities and large-area graphene films from graphene oxide solutions [28–31], but there are still limitations in accurately controlling the number of graphene layers and defect density [32, 33]. Top-down approach is not suitable for remote epitaxy due to small area and randomness of the graphene thickness which cannot be controlled at all. Thus, bottom-up approaches in which graphene is formed through the chemical bonding of carbon atoms onto a target substrate have been extensively investigated instead.

#### Bottom-up approach

Among the various substrates where sp^2^ carbon bonding is created, metal substrates such as copper [34–41], nickel [42–45], Pd [46] and Ir [47] are representative platforms from which graphene can be formed easily and quickly over the entire area through surface-catalyzed chemical vapor deposition (CVD) process. However, graphene grown on metal substrates not only has various defects including grain boundaries and wrinkles, but also has the challenge of precisely controlling the thickness of graphene over the entire substrate area [48]. The recently proposed Cu-Ni alloy substrate provides high-quality monolayer graphene with extremely decreased the wrinkle density, presenting a superior electric conductivity [49, 50]. In addition, diffusion-assisted synthesis (DAS) is suitable to fabricate large-area graphene on the desired substrate at low temperature [51, 52]. Also, research on growing single-layer graphene on various substrates using a bottom-up approach and then exfoliating or etching to grow multi-layer graphene has been conducted. This method produces large and densely packed graphene structures that offer the advantages of both approaches [53–55].

Recently, orientation-assisted growth of graphene on wafer-scale substrates excluding metal seed layers have been spotlighted due to the effective control of defects and wrinkles. A hydrogen-terminated germanium (110) surface provides a suitable platform for the catalytic reactions, forming high-quality single-crystal graphene via the low-pressure CVD (LPCVD) process [56–59]. In addition, highly oriented monolayer graphene was achieved on a c-plane sapphire wafer using an electromagnetic induction heating CVD method [60]. Compared to the growth method using a carbon-containing gas source, the SiC surface is transformed into pure carbon bonding by the thermal desorption of Si atoms under high temperature, resulting in single-crystal graphene via CVD [61–65] and MBE [66–69] techniques. These novel graphene growth techniques are highly desirable for remote epitaxy due to their thermodynamically limited growth mechanism which allows precise engineering of the graphene properties to be transferred onto single-crystalline substrates.

### TMDCs

Compared to graphene composed of a single layer of carbon, transition metal dichalcogenides (TMDCs) such as MoS_2_, MoSe_2_, WS_2_, and SnS_2_ are a class of 2D materials with stoichiometry MX_2_ in which a transition metal (M) atomic plane is sandwiched between two layers of chalcogen atoms (X). Due to their unique crystal structure, TMDCs exhibit tunable electronic and optical properties depending on chemical composition and the number of layers, which are actively applied in various future nanotechnology fields [14]. Similar to graphene exfoliation using adhesive tape, the layered structure of bulk TMDCs with strong in-plane bonding and relatively weak out-of-plane bonding can be separated into atomically thin 2D materials by physical exfoliation [70–75]. In addition, chemical/solvent exfoliation of bulk TMDCs produces 2D nanosheets with sub-micrometer size [76–80]. However, both exfoliation methods have critical limitations in fabricating large-area 2D materials and controlling their precise thickness. Thus, it is necessary to directly grow TMDCs on rigid substrates by the chemical reaction between transition metals and chalcogen elements for large-area and high-quality 2D materials.

The solution-based chemical synthesis method has the advantages of being able to get a large amount of various TMDC nanostructures including sheet, particle, and films, but it is difficult to obtain a large-area single-crystalline thin film with an accurate MX_2_ composition [81–83]. Vapor-phase growth process including CVD method is an innovative alternative that can overcome these limitations [84, 85]. Since layered 2D TMDCs are formed sequentially at high temperature through the chemical reactions based on vapor-phase precursors [86–89] and/or solid-phase precursors [90–95], it is possible to obtain high-purity and large-area 2D materials on the target substrates, as well as to control the morphology, crystallinity, and defect density of TMDCs. Although the use of TMDC for remote epitaxy has not been demonstrated much so far, it is theoretically possible to use TMDC as a 2D interlayer material as it is thin enough for materials with high ionicity such as complex oxide materials [13]. In a recent paper, the possibility of oxide remote epitaxy was shown of a three-dimensional material with a high degree of ionicity on MoS_2_, which is one of the TMDC materials [96, 97].

### Other 2D materials

Two-dimensional hexagonal boron nitride (h-BN) connected by strong sp^2^ covalent bonding, an isomorph of graphene with similar lattice parameters [98], is receiving great attention in future electronic devices due to excellent mechanical properties [99, 100], chemical stability [101, 102] and optical properties [103]. Similar to other 2D materials, the layered structure bulk h-BN can be separated into monolayer or 2D structures with a thickness of several nanometers by mechanical and chemical exfoliation [104–117]. However, top-down approaches to obtain h-BN are not suitable for controlling the exact number of layers and forming large-area 2D materials onto the target substrates. The metal substrates such as Cu [99, 118–126] and Ni [126–133] serve as appropriate seed platforms to artificially grow large-area h-BN via CVD process, and monolayer h-BN is grown to a size of 7500 μm^2^ with controlled nucleation density on the Cu-Ni alloy [134]. In addition, various methods such as LPCVD [135], PVD [136–139] and PLD [140, 141] have been proposed to fabricate large-area high-quality h-BN. Although crystalline 2D materials show excellent properties by controlling thickness, doping, and grain size, there are still remaining issues such as substrate dependence and high process temperature to be addressed. Compared to crystalline 2D materials, large-area amorphous h-BN [142–147] and amorphous graphene [148–155] formed independently of the substrate at low temperatures show high thermal stability and excellent dielectric properties at atomic thickness, which expands the application of 2D materials. To date, h-BN has been extensively used as a seed layer for vdW growth of GaN, even on amorphous substrates.

## Methods of producing 2D materials coated single-crystalline wafers

Non-conventional vdW and remote epitaxial growth techniques are typically performed on single-crystalline substrates or even amorphous substrates coated with 2D materials. For remote epitaxy, single-crystalline substrate is necessary whereas for vdW epitaxy, any crystalline substrate typically works as long as the surface is atomically smooth. For remote epitaxy, the 2D materials play a key role in the crystallographic registration and layer transfer of the grown epitaxial layer. In this section, methods for producing single-crystalline wafers coated with 2D materials such as transfer, direct growth, and in-situ 2D growth will be discussed.

### Transfer

This approach involves growth or deposition of 2D materials on one substrate, then transferring the 2D material to another single-crystalline substrate for epitaxial growth. This section mainly focuses on the transfer of graphene, a representative 2D material that is almost exclusively used for remote epitaxy. Typically, epitaxial graphene via graphitization of single-crystalline SiC wafer or growth through CVD on a metal catalyst foil are mainly used.

**Fig. 2:**
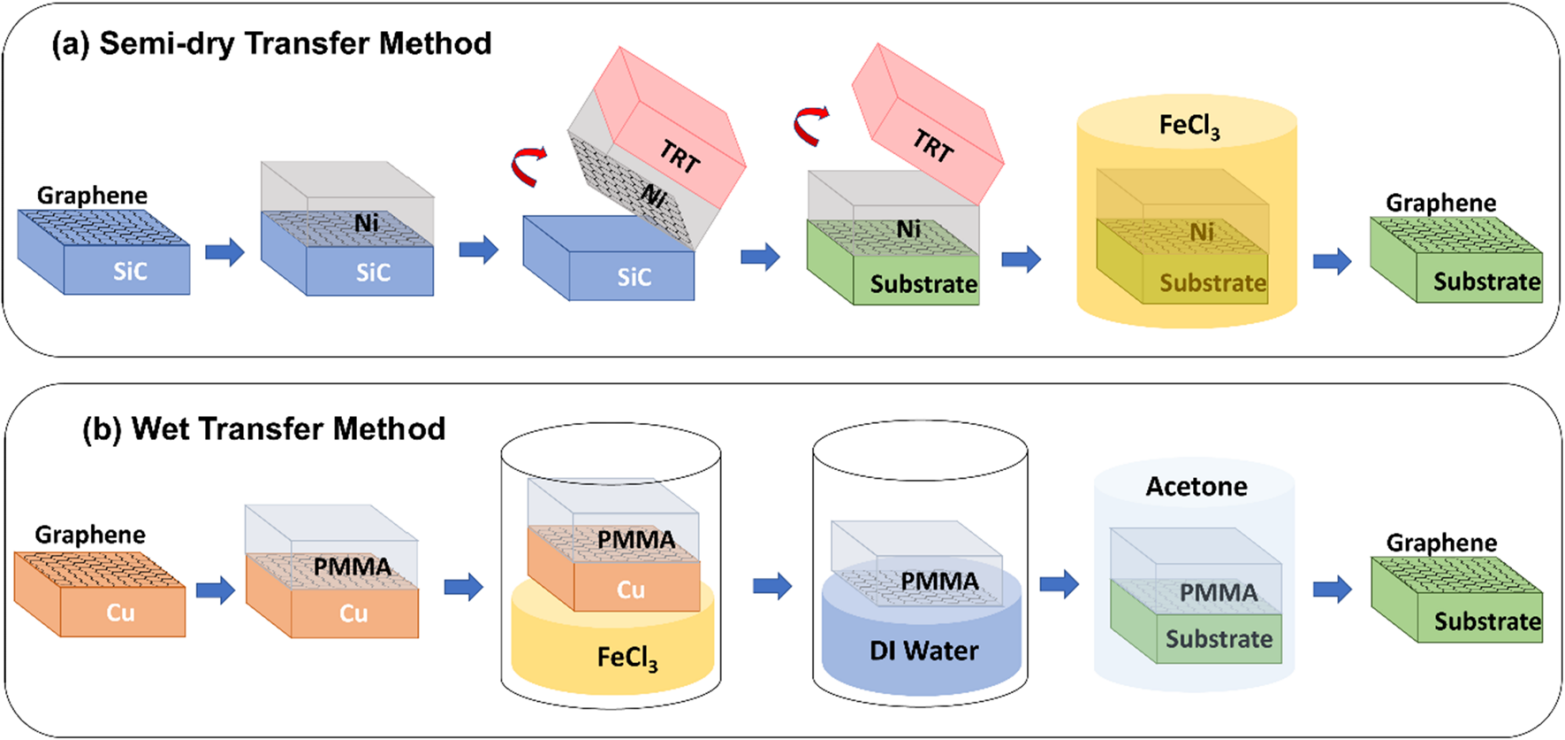
The schematic illustration of graphene transfer methods. **a** Semi-dry transfer method, **b** Wet transfer method

Epitaxial graphene is formed by sublimation of silicon and the rearrangement of carbon in a high temperature annealing of SiC wafer over 1500°C, called graphitization, and is one of the most reliable methods to obtain wafer-scale single-crystal graphene [156, 157]. The graphene can then be transferred to any arbitrary substrate without significant degradation of quality via a semi-dry transfer method (Fig. 2a) [62, 158]. The process begins with the deposition of a stressor layer, such as Ni, on graphene. An evaporation is recommended for stressor deposition rather than sputtering in order to avoid damage caused by ion bombardment on the graphene. Then, after attaching the thermal release tape (TRT) as a handling layer, the TRT/Ni/graphene stack is released from the SiC wafer by the mechanical exfoliation. The exfoliated stack is directly transferred to the target substate, and the TRT is removed through thermal release at around 120 °C using a hotplate. Finally, the stressor layer is etched using a Ni etchant, such as FeCl_3_, leaving only graphene on the target substate. The high-quality epitaxial graphene coated single-crystalline substrate can be obtained by using graphitization of SiC wafer and semi-dry transfer, but expensive SiC wafer and high-temperature processes for graphitization are required, and some defects on the graphene may occur during the transfer process.

On the other hand, large-scale graphene can be grown on a metal catalyst through CVD. In particular, Cu foil is a well-known metal catalyst for graphene growth, enabling the synthesis of large-area graphene at a relatively low temperature [36, 159]. The growth of graphene is mainly performed by flowing carbon precursors such as methane and ethane. The graphene grown on Cu foil can be transferred to the substrate of interest via a wet transfer technique (Fig. 2b) [160, 161]. In this process, first, the surface of the graphene to be transferred is spin-coated with a polymer such as poly(methyl methacrylate) (PMMA) to form a semi-rigid supporting layer. Then, the PMMA/Graphene/Cu foil stack is placed on top of a Cu etchant, such as FeCl_3_, to etch the Cu foil. This step may take several hours depending on the size and shape of the Cu foil. After the Cu foil is etched, the PMMA/graphene stack is rinsed with DI water to remove etchant residues, then transferred by scooping to the target substrate. Finally, the supporting layer is removed using acetone, leaving only graphene on the substrate of interest. In addition to graphene, other 2D materials can also be grown on metal catalysts through CVD. For example, h-BN, a polar 2D material, can be synthesized over a large-area on metal foil [124]. Similar to graphene, it can also be transferred to a target substrate via wet transfer process [162]. As an alternative to etching the metal catalyst, a way to reuse the catalyst foil through dry transfer using a polymer layer has also been proposed [163, 164]. In this method, large-area 2D materials can be obtained at a relatively low temperature without an expensive single-crystalline substrate, but may require an additional process to remove organic residues caused by the polymer used in the transfer process.

### Direct growth

As an alternative to the transfer method, 2D materials can be directly grown on the surface of the substrate on which remote epitaxy will be performed. In this method, various 2D materials, including graphene, TMDC and h-BN, can be formed directly on a semiconductor or insulator substrate without the need to form graphene on a single-crystalline SiC wafer or metal catalyst. The CVD method is mainly used for the direct growth. The 2D materials on the substrate are grown by injecting precursors in the form of gas or solid into a tube furnace. For example, direct growth of 2D materials, including graphene, TMDC and h-BN, has been reported not only on SiO_2_/Si [86, 165], Ge [56], sapphire [60, 135, 166], and SiC [63], but also on SrTiO_3_ substrate [167], a complex-oxide material that is drawing significant attention for next-generation electronic and photonic devices thanks to their functional properties including high-κ dielectrics [168, 169], ferroelectric [2], magnetism [170] and superconducting [171].

This technique is highly scalable method that can form 2D materials on substrates without leaving behind any tearing or residues occurred in the transfer process. However, it is still difficult to precisely control the thickness and uniformity of the grown 2D materials. In addition, a relatively high growth temperature might be needed since there is no metal catalyst that reduces the energy required for CVD growth of 2D materials. This makes it difficult to grow 2D materials on thermally weak substrates that cannot withstand the CVD growth temperatures of 2D materials [172]. To overcome these drawbacks, for example, 2D layer thickness control and removal of clumps of carbon atoms through atomic layer etching (ALE) technique [55], low-temperature 2D growth technology enabled by plasma [144, 152] have also been reported. Thus, although direct growth of graphene on the growth substrate is likely the most attractive option, a novel technique to control the thickness of the grown graphene layer, such as ALE, will become more important for the future of remote epitaxy.

### In-situ 2D growth

**Fig. 3:**
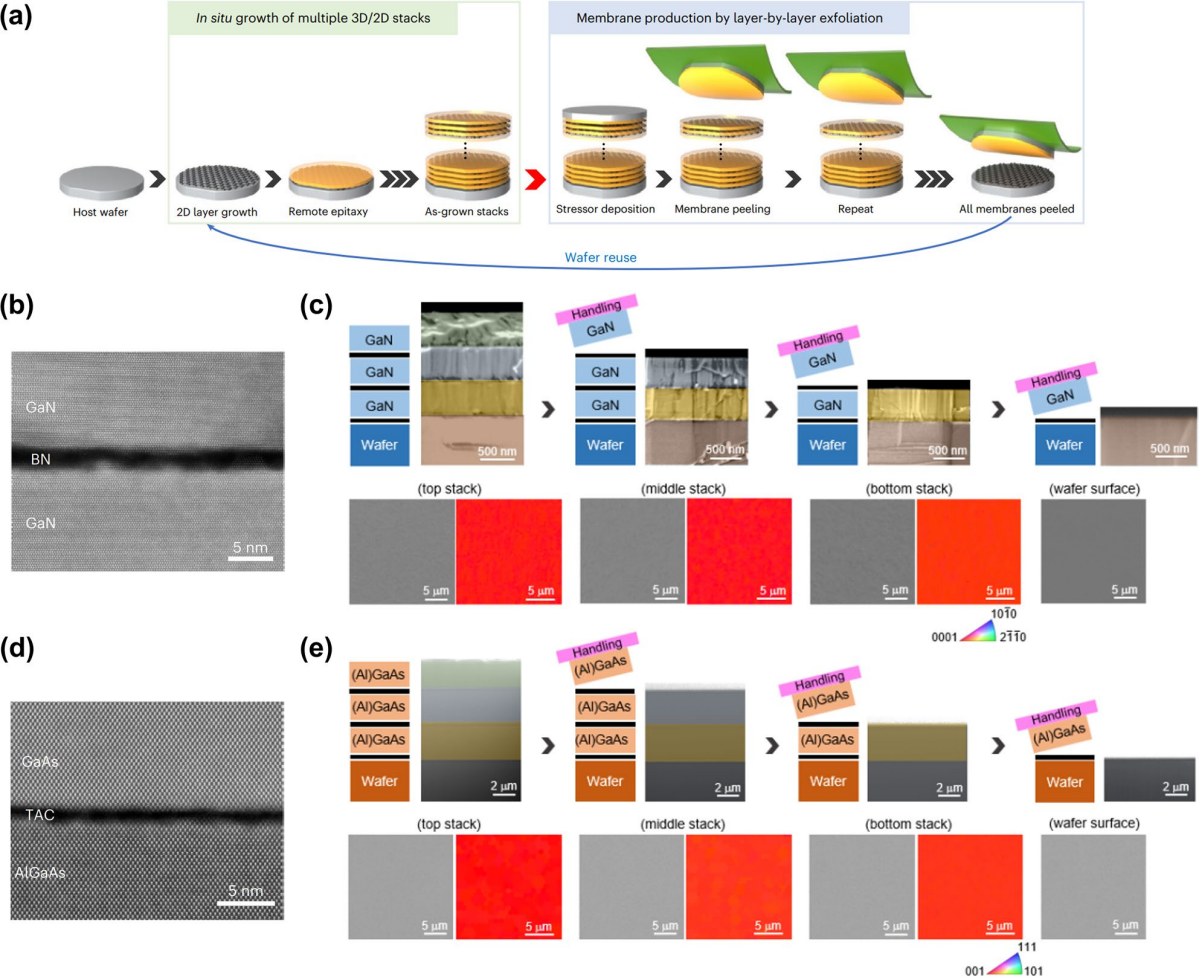
Multiplication of freestanding membranes via in situ growth **a** The schematic illustration of membrane production process via in situ growth. **b** Cross-sectional TEM image of remote epitaxially grown GaN on BN/GaN. **c** False-color cross-sectional, Plan-view SEM and EBSD map of as-grown and after exfoliated GaN. **d** Cross-sectional STEM image of remote epitaxially grown GaAs on TAC/AlGaAs/GaAs. **e** False-color cross-sectional, Plan-view SEM and EBSD map of as-grown and after exfoliated GaAs. Figure reproduced from ref. [173], Springer Nature Ltd

In-situ 2D growth is a recently proposed method of direct growth of 2D materials on a substrate in the epitaxy systems to grow 2D materials *in situ* with epitaxy [173]. The core of this technology is direct growth of 2D materials in epitaxial equipment, enabling efficient epitaxy without additional steps to form 2D materials on wafers, such as 2D transfer, loading/unloading, and ramping/cooling. In addition, alternating stacks of 2D materials and epitaxial layers can be grown in a single run, and multiple wafer-scale freestanding single-crystalline membranes can be obtained with high throughput via 2DLT [3] (Fig. 3a). The process begins with direct growth of 2D materials on a host wafer in the epitaxial system. The boron nitride (BN)and thin amorphous carbon (TAC) were grown on GaN and GaAs wafers using conventional epitaxy tools, MBE and MOCVD, respectively. Then, epitaxy is performed on the 2D-coated single-crystalline substrate, and multiple stacks of alternating 2D/epilayer are formed by repeating 2D growth and remote epitaxy. After that, a stressor layer is deposited on the topmost epilayer, and layer-by-layer peeling enabled by 2DLT is repeated to harvest multiple freestanding single-crystalline membranes. For proof of concept, three stacks of 2D/epilayer of GaN and GaAs were grown, and X-ray diffraction (XRD) spectrum showed that all epitaxial layers were grown along the crystallinity of the substrate (Fig. 3b and d). In addition, cross-sectional and plan-view scanning electron microscope (SEM) images and EBSD maps showed that multiple freestanding single-crystalline membranes could be harvested from a single wafer (Fig. 3c and e).

## Interaction between growth technique, 2D material, and substrate during various epitaxy techniques

In the following sections, we will discuss what type of epitaxy has been realized utilizing 2D materials on various substrates, and how to implement remote epitaxy successfully for various growth conditions and methods.

### 2D material on Non-nitride substrate

**Fig. 4:**
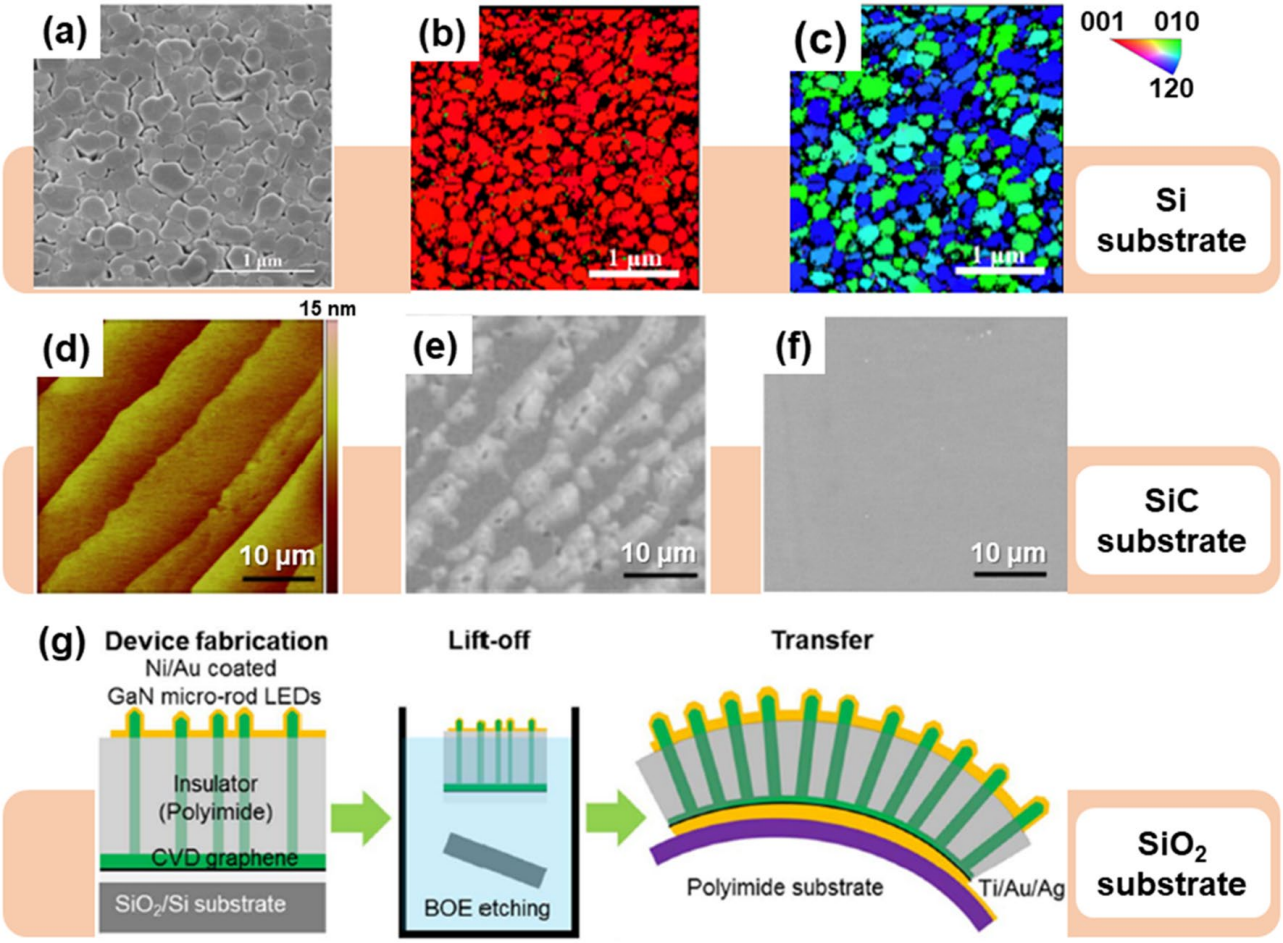
Epitaxy of graphene on non-nitride substrate. **a** The tilted-view SEM image of AlGaN nanorods on graphene/Si (111). **b** the normal-direction EBSD inverse pole figure (IPF) image of on graphene/Si (111). **c** the transverse-direction EBSD IPF image of AlGaN nanorods on graphene/Si (111). Figure reproduced from ref. [174], MDPI. **d** AFM image of the surface of a graphitized SiC substrate. **e** Plan-view SEM images of GaN films grown on graphene by one-step growth at 1100 ºC, and **f** modified two-step growth. Figure reproduced from ref. [8], Springer Nature Ltd. **g** A schematic illustration of the fabrication process for vertical structure micro-rod LEDs. Figure reproduced from ref. [175], AIP Publishing

To date, Si has been the most widely used semiconductor substrate due to its extremely low price and ease of fabrication. In fact, due to its extreme cost effectiveness, Si is even used as a substrate to grow GaN and its alloys. For example, Araki et al. reported c-axis oriented GaN growth on Si (100) using plasma-excited molecular beam epitaxy (PA-MBE) where graphene was introduced as an interlayer [176]. They showed that due to the unique structure of the graphene lattice, c-plane oriented GaN growth is possible even on substrates with non-epitaxial relationships such as Si. However, although out-of-plane alignment was possible, in-plane alignment was shown to be rather difficult due to the polycrystalline nature of the graphene used in the study. Since Si (111) has an epitaxial relationship with the hexagonal GaN structure on the surface, it has been mainly used for GaN microrod growth rather than planar films [174, 177–180]. In a report by Ren et al., it was shown that it is possible to grow AlGaN nanorod LEDs on graphene coated Si (111) substrates via MOCVD [174]. Figure 4a is the tilt-view SEM image of AlGaN nanorod LEDs on graphene/Si (111). Afterward, EBSD analysis shows that c-axis orientation is well matched, but in-plane alignment is not achieved (Fig. 4b, c). Zheng et al. compared InGaN nanorod growth (via PA-MBE) patterns in cases where monolayer graphene and trilayer graphene were used as interlayers [179]. In particular, it was reported that the trilayer graphene had higher adsorption energy and lower migration energy, which resulted in better nucleation in the initial growth stage, and affected the increase in nanorod density. These studies clearly show the possibility of epitaxial growth of compound semiconductors on graphene coated substrates. However, the mechanism of growth is still not fully understood whether the epitaxial growth is by vdW epitaxy or remote epitaxy.

SiC is also a popular substrate for vdW or remote epitaxy growth of GaN or other wide bandgap materials, as it is possible to form wafer-scale epitaxial graphene without the need to go through the wet graphene transfer method. High-quality epitaxial graphene with precise thicknesses can be formed on SiC through Si sublimation and graphitization at very high temperatures (~ 1800 ºC) [8, 61, 181–185]. Epitaxial graphene is one of the best suitable buffer layer for implementing vdW or remote epitaxy by utilizing the naturally formed vdW honeycomb lattice surface of the graphatized SiC. Kim et al. utilized the advantage of GaN nucleation (Fig. 4e) at the step edge of epitaxial graphene on SiC (0001) (Fig. 4d) to produce single-crystalline GaN film growth (via MOCVD) and even demonstrating a blue LED [8]. They reported this growth method direct vdW epitaxy and then successfully separated the grown GaN through a Ni stressor and showed transferable potential. Later, more detailed research revealed that this growth method was actually remote epitaxy and not vdW epitaxy [61].

SiO_2_ is formed on the Si surface through natural oxidation or deposited to a thickness of hundreds of nanometers and has been mainly used as an insulator and a sacrificial layer (Fig. 4g) [25, 175, 186–189]. In 2010, Chung et al. grew a transferable GaN film on ZnO-coated graphene. Subsequently, they transferred it to the foreign substrate through mechanical lift-off to implement the device, showing the possibility of exceeding the limitations of conventional epitaxy technology based on direct bonding [186]. However, due to the low surface energy of graphene, O_2_ plasma treatment was required to form a dangling bond on the graphene surface for the attachment of adatom during the growth process. Li et al. also grew the GaN epitaxial layer by introducing O_2_ plasma treatment on the graphite surface, revealed the growth mechanism, and suggested the possibility of making photoelectric devices through high-purity graphite [25]. Except for a specific case (on SiC substrate), these reports have primarily implemented vdW epitaxy using graphene lattice characteristics on the non-nitride substrates.

**Fig. 5:**
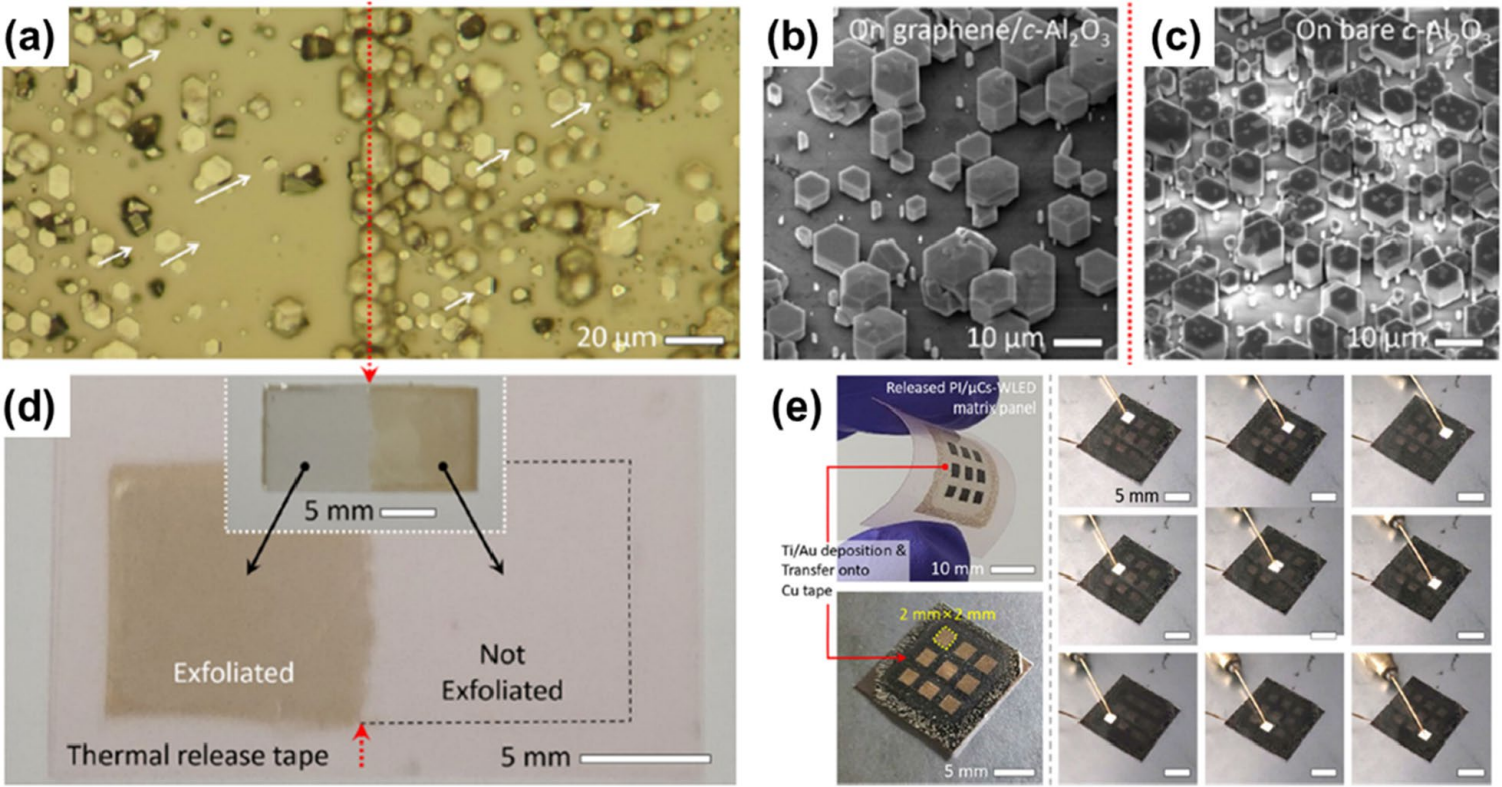
Remote epitaxy of 2D materials on sapphire (Al_2_O_3_) substrates and the principle. **a** Optical microscopic image of GaN microcrystals (μCs) taken at around the boundary between graphene/Al_2_O_3_ and bare Al_2_O_3_ surfaces. Based on the red dotted line, the left is on graphene/Al_2_O_3_ and right is on bare Al_2_O_3_. Tilted-view SEM images of GaN μCs grown on **b** graphene/Al_2_O_3_ and **c** bare Al_2_O_3_. **d** Photograph of the thermal release tape after the delamination of polyimide-encapsulated GaN μCs. The inset of **d** shows a photograph of the substrate after the exfoliation process. **e** Flexible white μCs-LED matrix arrays fabricated by forming patterned electrodes. Figure reproduced from ref. [190], Elsevier

Sapphire (Al_2_O_3_) substrates are relatively inexpensive compared to other substrates for GaN growth despite a lattice mismatch of about 16% with GaN [191]. However, a low-temperature buffer layer or superlattice is usually necessary to grow high-quality GaN layers on top of sapphire. In 2012, Choi et al. used the DAS method to directly coat a sapphire substrate with graphene and succeeded in the growth of GaN in one-step via MOCVD. In the initial state, it is revealed that GaN nucleation begins along the graphene ridge, allowing graphene to replace GaN buffer layers [51]. Similarly, Mun et al. compared the initial growth patterns of GaN using MOCVD through multi-stacked graphene buffer layers on sapphire and analyzed the stress relaxation effect of the grown GaN microstructure [192]. Zeng et al. attempted to grow AlN on the graphene buffer layer and reported that defects in the graphene layer play a crucial role in the AlN nucleation [193]. Similarly, Sarau et al. confirmed that controlling the defects in graphene during growth is possible through nitrogen doping on the surface of the graphene, which is advantageous for n-GaN rod formation [194]. Graphene defects were intentionally created through exposure to NH_3_ gas at high temperature or N_2_ plasma treatment. Other growth methods using graphene in various forms, such as multilayer, hybrid, and plasma-treated graphene, have been reported [194–198]. These methods, called vdW epitaxy or quasi-vdW epitaxy, were successful in membrane fabrication but had difficulties in exfoliation of the membrane off of the substrate. However, Jeong et al. was able to fabricate exfoliable GaN microrod LEDs on graphene coated sapphire without utilizing graphene defects. They reported "remote heteroepitaxy" and showed that the remote epitaxy technology can be applied to epitaxial growth of heterostructures (Fig. 5a–c). Subsequently, they were successful in transferred the fabricated GaN microrod LED to different substrates using TRT (Fig. 5d, e). Through these reports, they proved that c-axis oriented GaN growth (like on Si or SiO_2_ substrate) is possible via remote epitaxy producing well-aligned GaN micro-rod LEDs with in-plane alignment (Fig. 5a) [190, 199].

### 2D material on III-N substrate

Aluminum Nitride (AlN) is a material with a wurtzite structure and a lattice mismatch of ~ 2.4% with GaN. The polarity of AlN substrates is rather advantageous for improving the crystal quality of GaN growth, and is another good candidate for remote epitaxy [40, 200–205]. Zhang et al. succeeded in high-quality GaN growth, including low-threading dislocation through the modulation of graphene surface states by transferring graphene on sputtered AlN templates [200]. More recently, Liu et al. compared growth on a non-epitaxial substrate along with an AlN-deposited (via PVD) substrate and realized polarization-driven selective growth (OSG) through the growth pattern of AlN nuclei after graphene transfer [204]. As a result, they showed that single-crystalline GaN growth is possible on any non-epitaxial substrate by applying the OSG strategy. However, they found it was difficult to achieve in-plane alignment because the PVD-deposited AlN was polycrystalline.

**Fig. 6:**
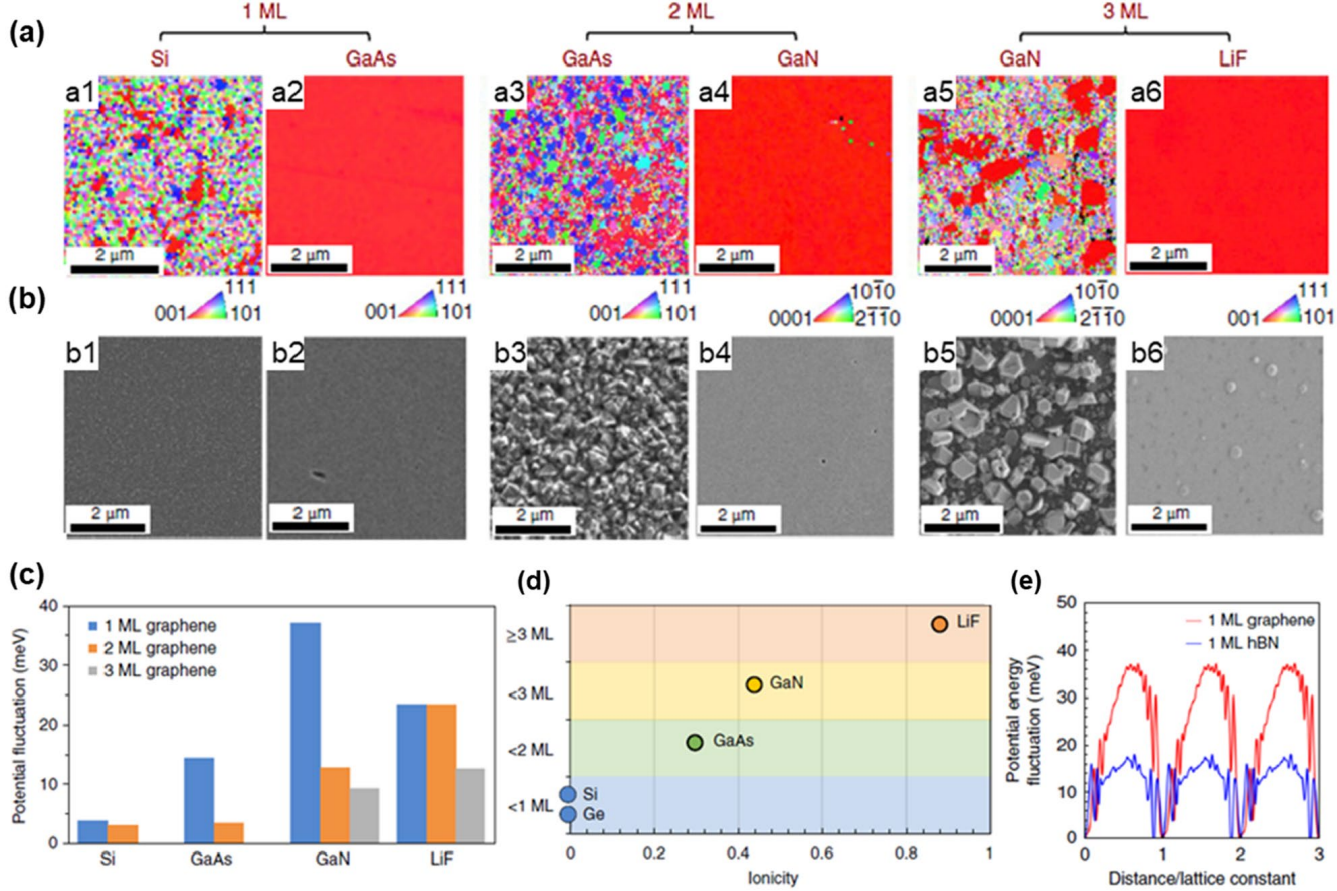
Remote epitaxy of 2D materials on III-N substrates and penetration distance of the potential fluctuations. Si/1-monolayer (ML)-graphene (Gr)/Si for **a1** and **b1**, GaAs/1-ML-Gr/GaAs for **a2** and **b2**, GaAs/2-ML-Gr/GaAs for **a3** and **b3**, GaN/2-ML-Gr/GaN for **a4** and **b4**, GaN/3-ML-Gr/GaN for **a5** and **b5**, LiF/3-ML-Gr/LiF for **a6** and **b6**. **a** EBSD of released surfaces. **b** Scanning electron microscopy morphology of as-grown surfaces. **c** Comparison of the effective potential energy fluctuation on 1-ML, 2-ML, and 3-ML graphene coated substrates. **d** The schematic of the remote interaction penetration depth depending on iconicity across groups IV, III-V and I-VII materials show that graphene transparency increases with material iconicity. **e** Potential energy fluctuation from the GaN substrate through 1-ML graphene and h-BN. Figure reproduced from ref. [13], Springer Nature Ltd

In 2018, Kong et al. carried out remote epitaxy (via MBE and MOCVD) and compared the possibility of remote epitaxy according to the polarity of the substrate for a total of four materials, Si, GaAs, LiF, and GaN (Fig. 6a, b) [13]. They found that for materials with stronger ionicity, remote epitaxy was possible even through multilayer graphene depending on the ionicity strength (Fig. 6c, d). However when h-BN was used instead of graphene, GaN was seeded by the h-BN and not the substrate due to the polarity of the h-BN itself, hindering the polarity of the substrate to penetrate through (Fig. 6e). Further research from the same group confirmed that the remote epitaxy phenomenon occurred in the same way for complex-oxide-based substrates. In addition, they reported that dry-transferred graphene is more effective than wet-transferred graphene since it produces less defects and residues during the transfer process [202].

Through these reports, we can conclude that the type of epitaxy is determined by the polarity of the substrate, the type of interlayer, the number of interlayer stacks, and the quality of layers. Thus, remote epitaxy is only observed in specific cases to date.

In the following section, we will provide an overview of recent publications on how to successfully realize remote epitaxy strategically, especially focusing on remote epitaxy of GaN via MOCVD.

### III-Nitride epitaxial growth on 2D layer

**Fig. 7:**
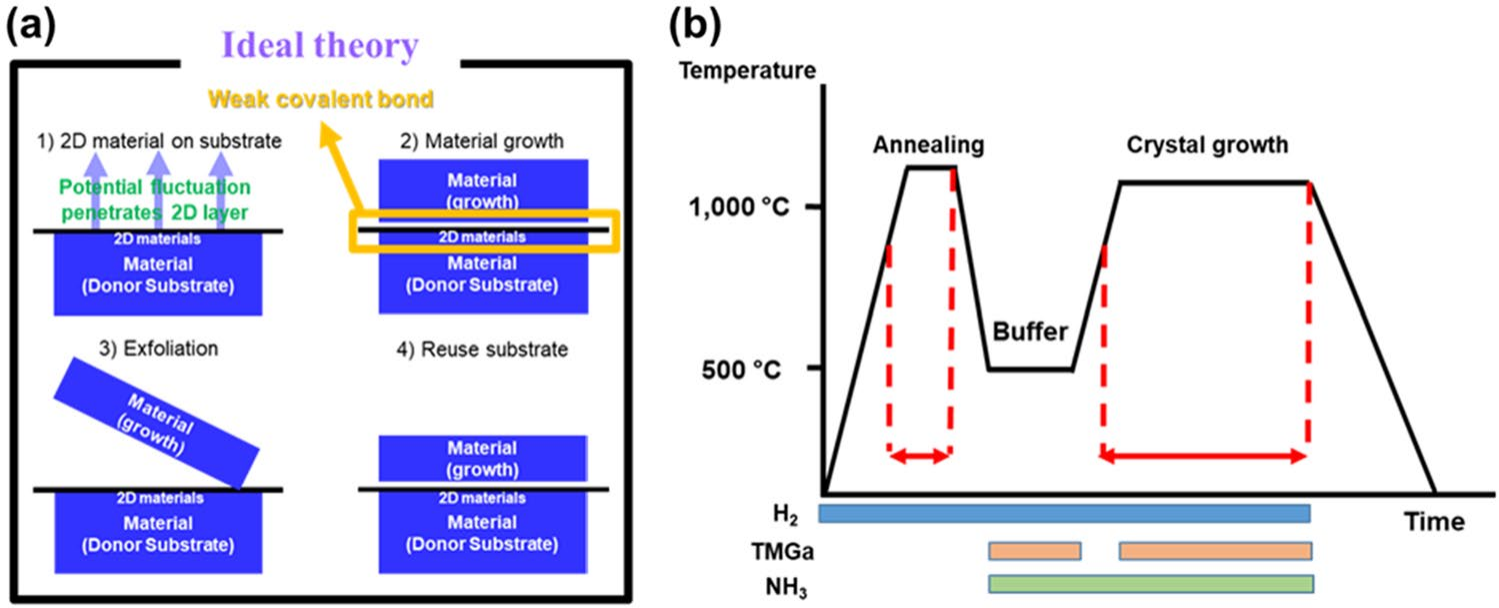
**a** Illustration of ideal theory of III-nitride growth on 2D layer. The weak covalent bond caused by an interfacial 2D layer between overgrown layer and the substrate allows the exfoliation of overgrown layer. Subsequently, a used substrate can be recycled for saving a cost. **b** Typical two-step GaN growth condition on c-plane sapphire substrate for high crystal quality GaN using MOCVD. This reveals that III-nitride growth conditions such as high temperature, and toxic gases (H_2_ and NH_3_) may change the properties of 2D layer

Recalling the mechanism of remote epitaxy as shown in Fig. 7a, it is possible to grow a single-crystalline thin-film on 2D material-coated substrates via the remote interaction of the substrate polarity through the 2D material with easy exfoliation of the thin-film off of the substrate [206]. Consequently, expensive substrates such as freestanding GaN, AlN, and silicon carbide could be recycled, ultimately resulting in significantly reduced device costs. To date, extensive results such as nitride growth on graphene or h-BN coated various substrates, showing exfoliation of overgrown layer and re-using substrate, have been reported [207–210].

Two-step growth including low-temperature buffer and crystal growth of nitride at high-temperature as shown in Fig. 7b is a universal technique that a lot of III -N growers are now using, which was discovered by Amano et al. in 1987 [211]. The interfacial buffer layer is the key factor determining single-crystalline III-N growth. As stated above, the condition of the interfacial 2D material is a key factor for successful remote epitaxy of III-N materials via MOCVD. One of the most important application for remote epitaxy is to be able to mass produce wafer-scale exfoliable GaN device films and re-using substrate. However, due to the relatively harsh nitride growth conditions in MOCVD, such as high temperatures and toxic gases, there is no guaranteed that 2D materials can remain undamaged. Since MOCVD reactor incubates gases such as TMGa, H_2_, and NH_3_ at high temperatures for growing III-nitride layers, understanding how these growth condition affect the 2D layers. A slight change in the condition of the 2D layer can critically change the success rate of remote epitaxy. Therefore, in this section, we will discuss the effect of ambient, temperature, and substrate on the 2D layer, providing a strategy for realizing successful remote epitaxy of III-nitride materials.

### Effect of hydrogen on 2D materials

Currently, the usage of H_2_ carrier gas is a common method for growing high-quality III-nitride alloys such as GaN, AlN, and AlGaN. Cho et al. found that using H_2_ carrier gas is more advantageous in decreasing the edge dislocation density compared to N_2_ carrier gas [214, 215]. N_2_ carrier gas is useful to grow a restricted alloyed material such as high indium content InGaN layer [216]. Since N_2_ has three covalent bonds that are not broken at the growth temperature of III-nitride, there is no concern of N_2_ reacting with the 2D material. Thus, using N_2_ may be desirable to realize remote epitaxy of InGaN. However, most nitride layers still need H_2_ carrier gas for obtaining high-quality films. Hence, it is necessary to discuss the effect of III-nitride carrier gas for remote epitaxy.

**Fig. 8:**
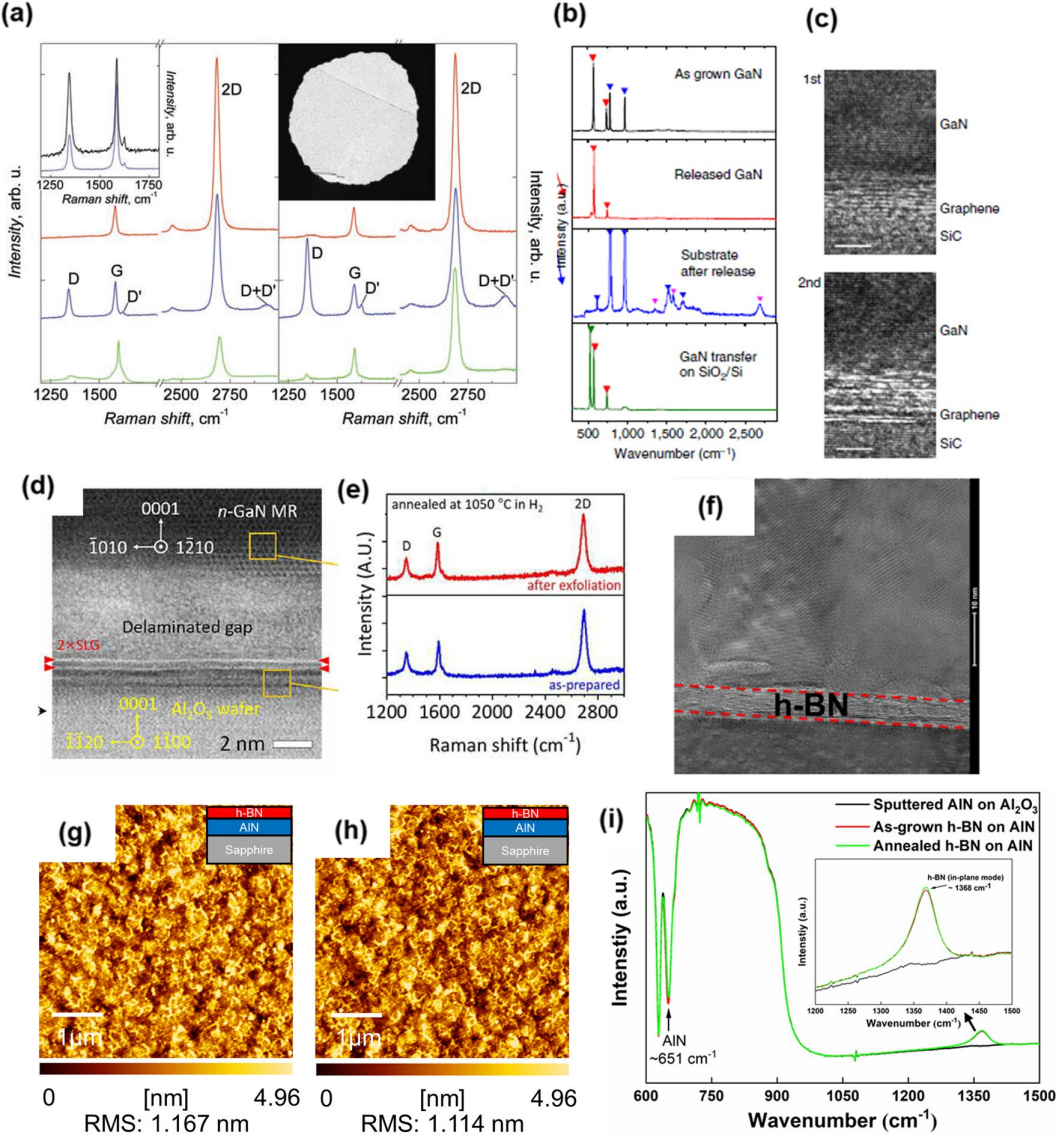
Effect of hydrogen on 2D materials. **a** Changes in Raman spectra of graphene caused by hydrogen. Left is Graphene on SiO_2_ and right is free-standing graphene. Red, blue, and green curves correspond to pristine, hydrogenated, and annealed samples, respectively. Figure reproduced from ref. [212], AAAS. **b** Raman spectra taken from an as-grown GaN film on graphene/SiC, released GaN films on the tape, remaining graphene after GaN release and GaN film transferred on SiO_2_ substrate. **c** HRTEM observed at the interface between GaN and SiC showing graphene remains after growth. Top image is GaN grown on a fresh graphene/SiC and bottom image is GaN grown on a reused graphene/SiC substrate. Figure reproduced from ref. [8], Springer Nature Ltd. **d** STEM image of GaN grown on graphene/c-sapphire confirming graphene remains. **e** Annealing test of wet-transferred graphene on c-sapphire at 1050 ºC ambient in H_2_. Figure reproduced from ref. [199], AAAS. **f** high-magnification CCD image of the interface between AlN and c-sapphire confirming the presence of h-BN layer. Figure reproduced from ref. [213], Elsevier. **g** 5 nm-thick h-BN layer as grown on AlN. **h** annealed h-BN on AlN at 1400 ºC ambient in H_2_. **i** FTIR spectra for confirming the presence of h-BN after annealing. Figure reproduced from ref. [203], RSC Publishing

In 2009, Elias et al. [212] reported the reaction between hydrogen atom and graphene layer. Hydrogenation produced D and D’ peak in the Raman spectra of graphene as well as a change in the lattice spacing between carbon atoms in case of both graphene on SiO_2_ and free-standing, as shown in Fig. 8a. The appearance of D, D’, and D + D’ peaks is attributed to hydrogenation which breaks the symmetry of the C–C sp^2^ bonds and forms C-H sp^3^ bonds. Furthermore, they found that the lattice constant of graphene, 2.46 Å, decreased as much as 5% after annealing, which was confirmed by TEM. After the annealing step, the Raman spectra recovered back to a pristine graphene state with strongly suppressed D, D’ and D + D’ peaks. Also, annealing led to the return of the original lattice constant. This result suggests that hydrogen atoms during annealing does not lead to significant changes in the characteristics of graphene. Additionally, hydrogen can also be used to remove organic PMMA residues on the graphene surface if the graphene is transferred using the wet transfer method [212]. Hydrogen gas also enables III-nitride growth on 2D material. In 2014, J. Kim et al. [8] reported GaN grown on graphene coated SiC substrates, where they optimized the growth condition in two steps. The first step is nucleation at 1100 ºC, and subsequently, the second step was carried out at 1250 ºC where hydrogen carrier gas was used. Figure 8b shows the Raman spectra of each step. After releasing the overgrown GaN layer, graphene D, G, and 2D peak were confirmed, indicating that the graphene can remain intact under hydrogen ambient. Clearly, the TEM image shown in Fig. 8c demonstrates that graphene is present at the GaN and SiC substrate interface. This observation suggests again that using hydrogen carrier is acceptable for remote epitaxy of GaN on graphene. Similar findings were also reported elsewhere [199]. GaN microrods were grown even on wet transferred either single or bilayer graphene on c-sapphire. The TEM observation (Fig. 8d) showed the presence of graphene after GaN growth as well as the delamination gap between overgrown GaN and c-sapphire. In addition, they tested high temperature annealing at 1050 ºC ambient in H_2_ for the graphene-coated substrate. Both single and bilayer graphene after annealing was confirmed to be intact by Raman spectra, as shown in Fig. 8e. Consequently, it can be concluded that wet-transferred graphene can survive under H_2_ ambient at the growth temperature of GaN. Figure 8 shows the potential for remote epitaxy while preventing graphene damage during the graphene growth process by wet transfer, but wet transfer of graphene to other substrates remains problematic. During the graphene transfer process, a native oxide is formed at the graphene and substrate interface, blocking graphene-mediated remote interactions. In contrast, dry transfer process produces a clean interface without oxidation, enabling successful atomic-level remote epitaxy through graphene, and exhibits more reliable remote epitaxy than the wet transfer process [11, 12].

As an alternative 2D material, boron nitride (BN), which is a III-nitride family, can be used for both vdW and remote epitaxy of III-nitride, with more stability than graphene, in the III-nitride growth environment. Figure 8f shows the interfacial hexagonal-BN (h-BN) layer between AlGaN/AlN and c-sapphire [213]. Prior to AlGaN/AlN growth, epitaxial h-BN was first conducted on c-sapphire at 1280 ºC in hydrogen ambient. Subsequently, AlGaN/GaN layer was grown on the as grown h-BN layer at 1140 ºC using hydrogen carrier gas. The observed high-resolution CCD image (Fig. 8f) for h-BN seems to be vivid and natural, confirming that AlGaN/AlN layer could be grown on h-BN using hydrogen gas without damage to h-BN layer [197]. Figure 8g, h show the atomic force microscopy (AFM) of the as grown h-BN on AlN and annealed one at 1400 ºC ambient in H_2_, respectively. Clearly, there seems to be no significant difference in the surface morphology and surface roughness before and after annealing. Furthermore, Fourier transform infrared (FTIR) spectra (Fig. 8i) obviously reveals that h-BN can survive not only under H_2_ ambient but also at high temperatures of around 1400 ºC, which is the temperature where AlN starts to decompose. These investigations of h-BN suggest that the 2D material containing a nitrogen atom, which is sufficiently robust under the III-nitride growth environment, may be desirable to realize vdW and remote epitaxy. There are many reported results for growing III-nitride on 2D materials under H_2_ carrier gas. This may indicate that H_2_ carrier gas alone is not the main cause of failed vdW or remote epitaxy.

### Effect of nitrogen on 2D materials

In fact, the properties of 2D materials alter when exposed to a nitrogen (N) environment [221, 222]. The effect of N on graphene results in not only a dangling bond but also vacancy sites. Since the surface energy of graphene is ultra-low, the direct growth of III-nitride materials on graphene is regarded as very challenging. To overcome this, artificially induced N-doped graphene has been adopted as an alternative solution to directly grow III-nitride on graphene.

**Fig. 9:**
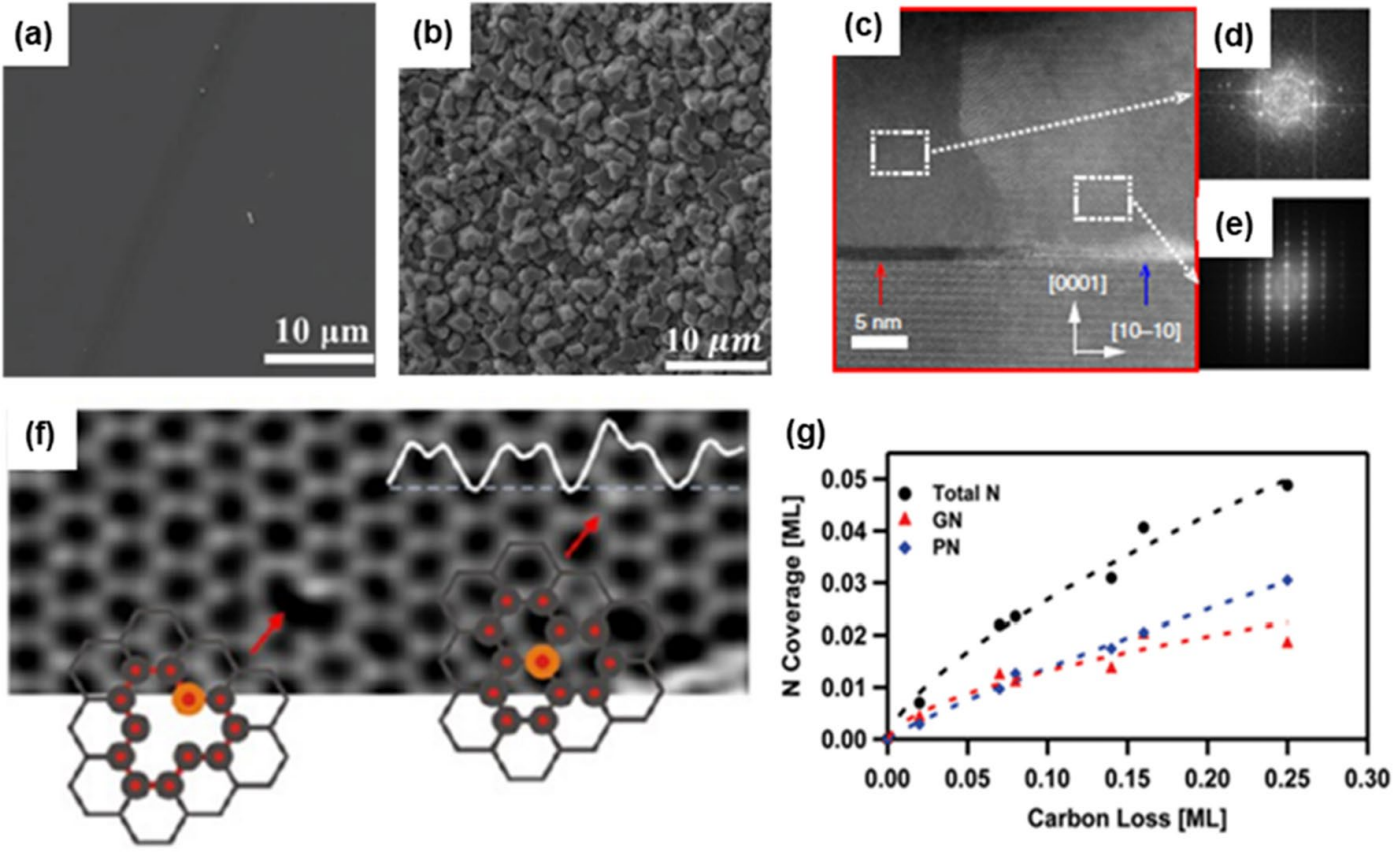
Plan-view SEM image of GaN growth on graphene/SiO_2_/Si substrate. **a** with NH_3_ annealing **b** without NH_3_ annealing. Figure reproduced from ref. [217], Wiley. **c** cross-sectional HAADF-STEM image of GaN on N-plasma treated graphene/SiC substrate. FFT pattern of AlN layer grown on **d** graphene regime, **e** etched graphene regime. Figure reproduced from ref. [218], Springer Nature Ltd. **f** atomic resolution HAADF-STEM image of graphene layer after N ion implantation showing N-atom adjacent to a vacancy and a substitutional N-atom. Figure reproduced from ref. [219], ACS Publications. **g** Demonstration that carbon loss of graphene is proportional to N coverage. Figure reproduced from ref. [220], ACS Publications

In 2017, Sarau et al. [194] reported that wet-transferred graphene on c-sapphire had N doping of approximately 11 atoms % in ambient NH_3_ at 1200 ºC for 10 min. NH_3_ decomposes by itself at high temperatures and provide the N atoms, which creates C-N covalent bonds. Subsequently, single-crystalline GaN layer (0002) can be grown on graphene/SiO_2_ annealed in NH_3_ environment due to the increased C-N bond number [217]. According to this work, NH_3_ annealing forms sp^2^ C-N (398.7 eV) and sp^3^ C-N (399.6 eV) bonding called pyridine N and pyrroline N, respectively. Figure 9a, b show the result of GaN growth on with and without NH_3_ treated graphene/SiO_2_, respectively. As explained above, since graphene has low surface energy, there is small chance that GaN can grow on graphene directly without invoking remote epitaxy. However, artificially induced C-N bonding is more active than sp^2^ C–C bonding. This C-N bonding triggers a formation of AlN seed layer on graphene, which allows single-crystalline GaN layer (0002) to be grown on graphene/SiO_2_. Instead of annealing in NH_3_, N_2_ plasma treatment has also been reported. Chen et al. showed that N_2_ plasma treatment increased the defect density, which yielded a facilitation of AlN nucleation site, thereby single crystalline AlN was obtained [196]. Another hypothesis is that the graphene layer is partially etched by N_2_ plasma treatment [218]. Figure 9c shows HAADF-STEM image observing the etched graphene region after N_2_ plasma treatment. Interestingly, the result of AlN growth differed depending on the graphene regions (i.e., unetched and etched). The selected area electron diffraction (SAED) demonstrated that AlN layer grown on unetched region was of polycrystalline (Fig. 9d) in nature, while on etched region was a single-crystalline (Fig. 9e).

Indeed, N-incorporated graphene is beneficial to increase the surface energy of graphene, which helps III atoms form III-N bond that aids in nucleation. However, previous studies pointed out that the graphene honeycomb lattice can break during N treatment (i.e., N-doped graphene structure) [220, 223, 224]. Bangert et al. [219] observed N-atoms adjacent to vacancy sites and substitutional N-atoms after low energy ion implantation of graphene (Fig. 9f). This implies that N atoms incorporated into graphene can create vacancy defects, replacing a carbon atom. Similar observation has been reported elsewhere [225]. Subsequently, carbon loss is proportional to the N coverage in graphene (Fig. 9g) [220]. These results indicate that the loss of carbon is related to the amount of inserted N, which results in vacancies or holes in the graphene lattice. Thus, on one hand, N treatment on graphene provides a chance that III-nitride is easily grown on graphene via defect or vacancy. On the other hand, N treatment may not be a desirable way for realizing III-nitride remote epitaxy since remote epitaxy requires a clean interface without any defects for interaction between the substrate and the growth material, which is a critical factor determining the successful exfoliation of the grown film. Also, the effect of NH_3_ that is used for III-nitride growth should be avoided or minimized during III-nitride growth since NH_3_ changes the performance of the graphene layer. Although there is no consensus for III-nitride remote epitaxy on graphene-coated substrates yet, especially III-nitride substrates, it is clear that N atoms affects the performance of graphene, resulting in reduced yield of III-nitride remote epitaxy. Therefore, a recipe protecting graphene during the epitaxy of III-N films via MOCVD needs to be developed moving forward.

### Stability of 2D materials on the substrates varying temperature.

**Fig. 10:**
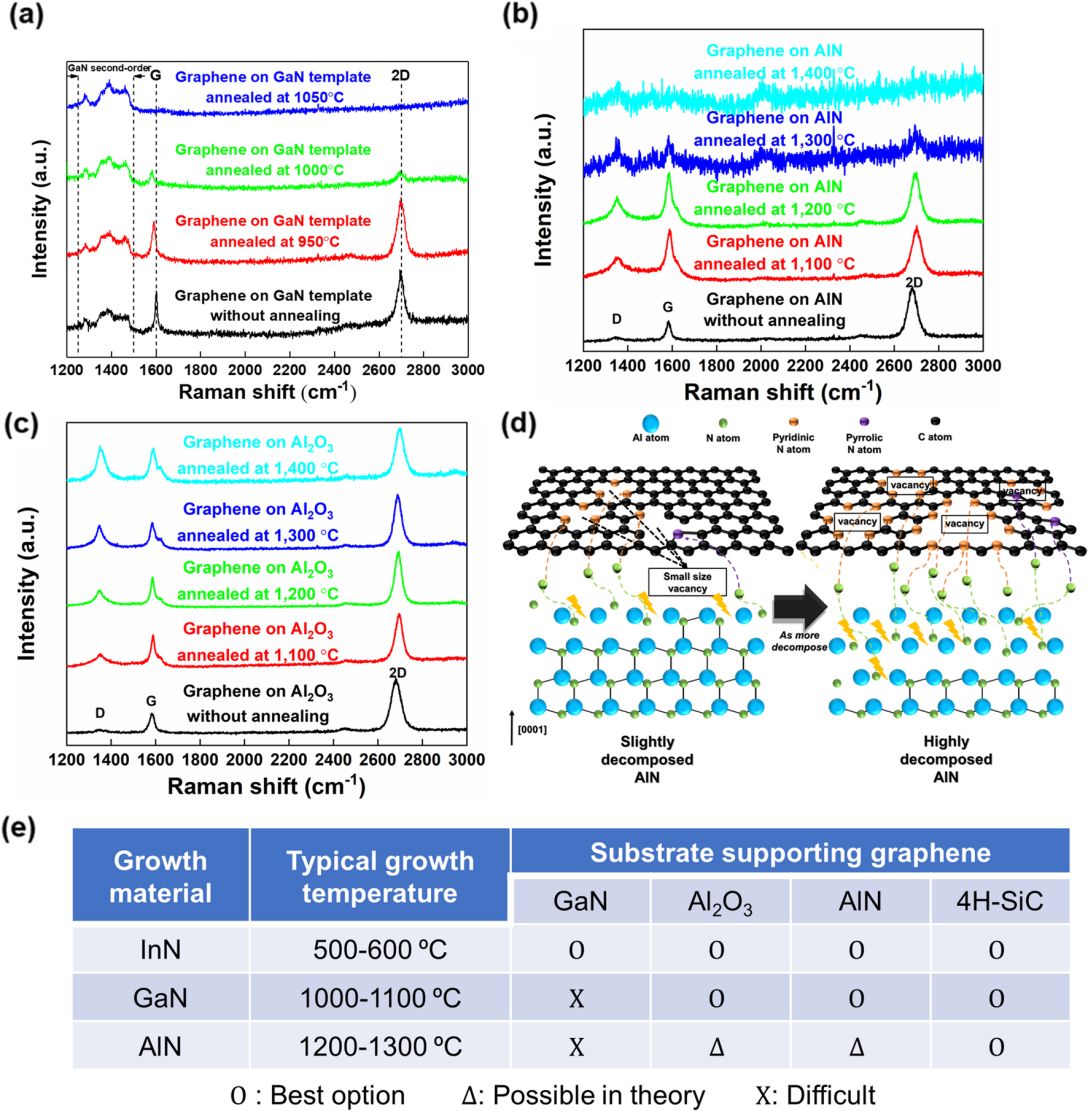
Stability of graphene on various substrates and at various temperatures. Raman spectra of graphene **a** on GaN at 950–1050 ºC. Figure reproduced from ref. [172], Wiley. **b** on AlN at 1100–1400 ºC **c** on Al_2_O_3_ at 1100–1400 ºC. **d** Illustration of occurrence of graphene-loss caused by generated nitrogen atoms through the decomposition of nitride-substrate. Figure reproduced from ref. [203], RCS Publishing. **e** The usage of desirable substrate for no graphene-loss and successful remote epitaxy under the III-nitride growth conditions

One challenge preventing remote epitaxy in III-nitride growth via MOCVD is the substrate decomposition issue. For high crystal quality of GaN, high temperatures over 1000 ºC is required [5, 226]. For homoepitaxy such as GaN on GaN, the decomposition of GaN substrate does not seriously affect growth results even at high temperature as well as H_2_ and NH_3_ ambient. However, since graphene is not guaranteed to survive in those conditions. According to Park et al. [172] the decomposition of the III-nitride substrate triggers graphene-loss. Furthermore, they compared the impact on 2D materials for non-nitride substrates and nitride substrates. The graphene coated GaN template was annealed at varying temperatures from 950 to 1050 ºC under N_2_ ambient (Fig. 10a). First, graphene was annealed on GaAs substrate for confirming which atom (N or As) is responsible for damaging graphene. It is deduced that N atoms are the main cause of graphene loss. Then, graphene-coated AlN and Al_2_O_3_ substates, substrates that are widely used in III-nitride growth, were systematically investigated under the same annealing conditions in H_2_ ambient (Fig. 10b, c) [203]. The H_2_ ambient was selected to investigate substrate decomposition at a relatively low temperature (assuming that H_2_ alone does not cause graphene damage). Similar to the results on GaN templates, no Raman peak was observed for graphene on AlN template over 1300 ºC, which is the starting AlN decomposition. On the other hand, graphene was observed on Al_2_O_3_ regardless of the annealing temperature, including temperatures higher than that of Al_2_O_3_ decomposition, even though the surface became rough with a lot of voids. This indicates that the decomposition of the substrate surface alone is not the main cause of complete graphene loss. Figure 10d describes the mechanism of graphene loss. It has been reported that carbon loss producing vacancy points and holes in the graphene structure occurs by N atoms, which is called N-doped graphene structure [220, 223, 224]. This, carbon-loss in graphene caused by N atoms, which seems inevitable due to the existance of N atoms in the substrate itself. We note that metal atoms such as Al, Ga, and In do not lead to carbon-loss, reported in the following studies [178, 182, 227]. Similar results for graphene loss on III-nitride substrate have been reported elsewhere [40, 205, 228].

## Summary and future outlook

In summary, for successful remote epitaxy, it is necessary to consider many factors such as choosing the most robust substrate at the material growth temperature and also growth conditions to minimize graphene damage during growth. Following this, several solutions can be reasonably deduced as shown in Fig. 10e. Typical growth temperatures of InN, GaN, and AlN are 500–600, 1000–1100, and 1200–1300 ºC, respectively. Meanwhile, it has been reported that the decomposition temperatures of InN, GaN, and AlN ambient in H_2_ are approximately 500, 700, and 1300 ºC, respectively [229–231]. Additionally, Al_2_O_3_ and SiC start to decompose at 1200 and 1700 ºC, respectively [232, 233]. Considering both growth temperature and decomposition temperature, the substrate having a higher decomposition temperature than growth temperature would guarantee the survival of the graphene during growth. Therefore, for III-nitride remote epitaxy, GaN on graphene/AlN (2.4% lattice mismatch) or AlN on graphene/AlN (0% lattice mismatch) may be a desirable approach when using MOCVD. Regarding GaN on graphene/GaN, it can be realized using MBE system that requires a low growth temperature of approximately 700 ºC, which temperature enables to avoid the decomposition of GaN substrate, if N-plasma is appropriately controlled [234]. Recent studies on modified hybrid MBEs [235] specifically designed for remote epitaxy show that engineering novel growth tools and techniques may be the best route to successful adoption of remote epitaxy on the industrial scale.

## Data Availability

The datasets used and/or analysed during the current study are available from the corresponding author on reasonable request.
