# Correction: Understanding the 2D-material and substrate interaction during epitaxial growth towards successful remote epitaxy: a review

**DOI:** 10.1186/s40580-023-00396-0

**Published:** 2023-10-21

**Authors:** Jongho Ji, Hoe-Min Kwak, Jimyeong Yu, Sangwoo Park, Jeong-Hwan Park, Hyunsoo Kim, Seokgi Kim, Sungkyu Kim, Dong-Seon Lee, Hyun S. Kum

**Affiliations:** 1 Department of Electrical and Electronic Engineering, Yonsei University, Seoul, South Korea; 2 School of Electrical Engineering and Computer Science, Gwnagju Institute of Science and Technology, Gwangju, South Korea; 3 Department of Nanotechnology and Advanced Materials Engineering, Sejong University, Seoul, South Korea; 4 Venture Business Laboratory, Nagoya University, Furo-Cho, Chikusa-ku, Nagoya, 464-8603 Japan

**Correction to: Nano Convergence (2023) 10:19** 10.1186/s40580-023-00368-4

Following publication of the original article [1], the authors noticed some errors.

In Sect. 4.3, “In-situ 2D growth”, and in caption of Fig. 3, the compound name "h-BN" should be changed to "BN (Boron nitride)" and "graphene" to be changed to "thin amorphous carbon (TAC)". In consistent with the corrections in text and figure caption, Fig. 3 is also updated.

**Fig. 3:**
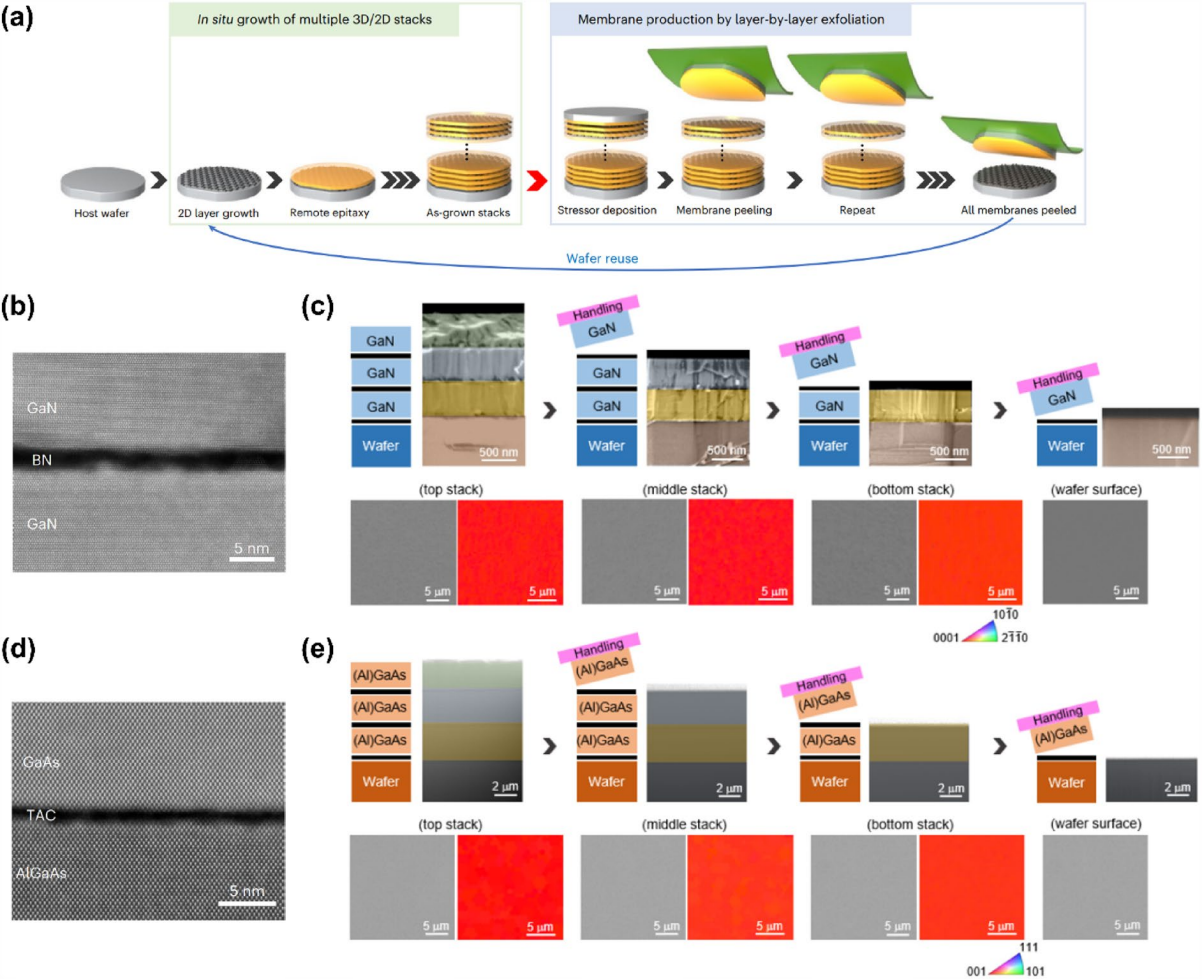
Multiplication of freestanding membranes via in situ growth **a** The schematic illustration of membrane production process via in situ growth. **b** Cross-sectional TEM image of remote epitaxially grown GaN on BN/GaN. **c** False-color cross-sectional, Plan-view SEM and EBSD map of as-grown and after exfoliated GaN. **d** Cross-sectional STEM image of remote epitaxially grown GaAs on TAC/AlGaAs/GaAs. e False-color cross-sectional, Plan-view SEM and EBSD map of as-grown and after exfoliated GaAs. Figure reproduced from ref. [173], Springer Nature Ltd

The original article [1] has been updated.
